# Targeting the Intestinal Microbiota to Prevent Type 2 Diabetes and Enhance the Effect of Metformin on Glycaemia: A Randomised Controlled Pilot Study

**DOI:** 10.3390/nu12072041

**Published:** 2020-07-09

**Authors:** Talia Palacios, Luis Vitetta, Samantha Coulson, Claire D. Madigan, Yan Y. Lam, Rachel Manuel, David Briskey, Chelsea Hendy, Ji-Nu Kim, Thomas Ishoey, Maria J. Soto-Giron, Eric M. Schott, Gerardo Toledo, Ian D. Caterson

**Affiliations:** 1 The Boden Collaboration for Obesity, Nutrition, Exercise and Eating Disorders, Charles Perkins Centre, University of Sydney, Sydney, NSW 2006, Australia; claire.madigan@hotmail.com (C.D.M.); chelsea.hendy@sydney.edu.au (C.H.); ian.caterson@sydney.edu.au (I.D.C.); 2 Faculty of Medicine and Health, University of Sydney, Sydney, NSW 2006, Australia; luis.vitetta@sydney.edu.au (L.V.); samantha.coulson@sydney.edu.au (S.C.); 3 Medlab Clinical, Sydney, NSW 2015, Australia; 4 Faculty of Science, Health, Education and Engineering, University of the Sunshine Coast, Sippy Downs, QLD 4556, Australia; 5 Department of Biochemistry and Microbiology and New Jersey Institute for Food, Nutrition, and Health, School of Environmental and Biological Sciences, Rutgers University, New Brunswick, NJ 08901, USA; yan.y.lam@rutgers.edu; 6 School of Medical Sciences, University of New South Wales, Sydney, NSW 2052, Australia; r.manuel@unsw.edu.au; 7 School of Human Movement and Nutrition Sciences, Faculty of Health and Behavioural Sciences, University of Queensland, Brisbane, QLD 4072, Australia; d.briskey@uq.edu.au; 8 Solarea Bio Inc., Cambridge, MA 02142, USA; realkjw@gmail.com (J.-N.K.); tishoey@solareabio.com (T.I.); jsoto@solareabio.com (M.J.S.-G.); eschott@solareabio.com (E.M.S.); gtoledo@solareabio.com (G.T.)

**Keywords:** prediabetes, type 2 diabetes mellitus, metformin, intestinal microbiota, probiotics, short-chain fatty acids

## Abstract

Early treatment may prevent or delay the onset of type 2 diabetes mellitus (T2DM) in individuals who are at high risk. Lifestyle interventions and the hypoglycemic drug metformin have been shown to reduce T2DM incidence. The effectiveness of such interventions may be enhanced by targeting environmental factors such as the intestinal microbiota, which has been proven to predict the response to lifestyle interventions and play a part in mediating the glucose-lowering effects of metformin. Shifts in the intestinal microbiota “towards a more balanced state” may promote glucose homeostasis by regulating short-chain fatty acids’ production. This study aimed to investigate the safety and effect of a multi-strain probiotic on glycemic, inflammatory, and permeability markers in adults with prediabetes and early T2DM and to assess whether the probiotic can enhance metformin’s effect on glycaemia. A randomised controlled pilot study was conducted in 60 adults with a BMI ≥ 25 kg/m^2^ and with prediabetes or T2DM (within the previous 12 months). The participants were randomised to a multi-strain probiotic (*L. plantarum*, *L. bulgaricus*, *L. gasseri*, *B. breve*, *B. animalis sbsp. lactis*, *B. bifidum*, *S. thermophilus*, and *S. boulardii*) or placebo for 12 weeks. Analyses of the primary outcome (fasting plasma glucose) and secondary outcomes, including, but not limited to, circulating lipopolysaccharide, zonulin, and short chain fatty acids and a metagenomic analysis of the fecal microbiome were performed at baseline and 12 weeks post-intervention. The results showed no significant differences in the primary and secondary outcome measures between the probiotic and placebo group. An analysis of a subgroup of participants taking metformin showed a decrease in fasting plasma glucose, HbA1c, insulin resistance, and zonulin; an increase in plasma butyrate concentrations; and an enrichment of microbial butyrate-producing pathways in the probiotic group but not in the placebo group. Probiotics may act as an adjunctive to metformin by increasing the production of butyrate, which may consequently enhance glucose management.

## 1. Introduction

The prevalence of prediabetes is increasing worldwide, and more than 482 million may develop it by 2040 [1]. Prediabetes occurs when blood glucose levels are higher than normal but not high enough to make a diagnosis of type 2 diabetes mellitus (T2DM) [2]. Prediabetes is of concern, as people with this condition are at high risk of developing T2DM and complications—particularly cardiovascular disease, nephropathy [3], and retinopathy [4,5]. Lifestyle interventions that produce weight loss have been shown to reduce the risk of developing T2DM by 30–58% in subjects with prediabetes [6]. However, the effectiveness of lifestyle interventions in inducing weight loss as well as glucose management and the occurrence of T2DM is influenced by the composition and function of the individual’s intestinal microbiota [7,8,9], which has also been shown to mediate and enhance the efficacy of metformin [10,11,12]. As a consequence, the intestinal microbiota is postulated as a therapeutic target for prediabetes and early diagnosed T2DM.

Probiotics are defined as “live microorganisms which, when administered in adequate amounts, confer beneficial health effects on the host” [13]. In patients with prediabetes and T2DM, probiotic supplementation may improve metabolic and inflammatory markers by inducing the production of dietary and microbial-derived metabolites, such as short-chain fatty acids (SCFAs), secondary bile acids, and choline by-products that influence energy metabolism. SCFAs (acetate, butyrate, and propionate) have been shown to regulate energy intake, body weight, and glucose homeostasis [14,15,16] by stimulating the secretion of gut hormones (glucagon-like peptide-1 and peptide YY) in human studies [17,18,19]. Although there has been significant progress in identifying the pathways responsible for the formation of SCFAs in models of the human colon [20,21], little is known about the capacity of probiotics to influence the production of SCFAs in individuals with prediabetes and T2DM.

Metformin, a drug commonly prescribed for treating T2DM, is known to act by reducing hepatic glucose production, but recent evidence suggests that some of the antidiabetic effects of metformin are also associated with changes in the intestinal microbiome [11,22,23]. Genetic and metabolic analyses of serum and stool samples revealed that metformin regulates bile acid and potentially glucose metabolism by decreasing the abundance of *B. fragilis* and its bile salt hydrolase activity in the intestines of participants with T2DM [24]. It has also been reported that the use of metformin increased the abundance of pro-inflammatory bacteria and decreased SCFA-producing bacteria in women with T2DM [25]. Shifts in the intestinal microbiota may mediate some of metformin’s glucose-lowering effects, but can also promote adverse effects such as intestinal dysbiosis and gastrointestinal symptoms, which can alter patients’ tolerance and adherence to metformin [26]. In a crossover study [27], metformin-intolerant patients with T2DM were treated with either a prebiotic formulation or placebo, both in conjunction with metformin. The prebiotic plus metformin increased the tolerance to metformin and led to lower fasting glucose levels than the placebo combination. It is plausible that enhancing metformin’s therapeutic effect using microbiome modulators (i.e., prebiotics or probiotics) may improve glucose management in patients with T2DM.

This current study was conducted to evaluate the efficacy of an evidence-based multi-strain probiotic on glycaemia, intestinal permeability, and inflammatory markers in adults with prediabetes and early T2DM (less than one year from diagnosis) and to assess whether the probiotic had an additive effect on glycaemia in patients prescribed with metformin. We hypothesied that the increased production of butyrate induced by the multi-strain probiotic would regulate the metabolic and inflammatory responses and result in improved blood glucose management.

## 2. Materials and Methods

### 2.1. Design

Details regarding the design of this study, the recruitment process, the allocation and description of study participants, the schedule of visits, the data collection, and the power calculations have been published previously [28]. In brief, this was a pilot study, single site, randomised, double blind, placebo-controlled clinical trial conducted at the Charles Perkins Centre Royal Prince Alfred Clinic (CPC RPA Clinic) in Sydney, Australia. This study was carried out according to the Declaration of Helsinki; approved by the Sydney Local Health District Human Research Ethics Committee, RPA Hospital, Sydney, Australia (X14-0369 & HREC/14/RPAH/492); and registered on the Australian New Zealand Clinical Trial Registry (ACTRN12613001378718). Written informed consent was obtained from the participants before enrolment.

### 2.2. Subjects

The participants were eligible for the study if they met the following criteria: aged ≥18 years; BMI ≥ 25 kg/m^2^; had prediabetes or T2DM diagnosed within the previous 12 months as per the American Diabetes Association criteria [29]; treated by diet alone or diet plus metformin; not diagnosed with gastrointestinal disorders; not taking anti-obesity drugs or blood glucose-lowering medications (other than metformin); not taking antibiotics and/or dietary supplements, including probiotics, for four weeks prior to commencing the study; and willing to adhere to the study protocol for the duration of the study.

### 2.3. Intervention

Sixty eligible participants were randomised to receive lifestyle advice and two capsules twice per day of either a multi-strain probiotic or placebo (200 mg microcrystalline cellulose, 10 mg silica, and 10 mg magnesium stearate per capsule) for 12 weeks. Capsule counting at weeks six and 12 was used to assess the participant’s compliance to the study product. A participant was defined as non-compliant if they took less than 80% of the study product on both occasions.

Dietary and lifestyle advice was provided by a research dietitian who was blinded to the allocation of participants to the treatment groups. Identical information was provided to all the study participants, and the details have been previously reported [28]. The participants were asked to record their food intake and the total number of steps walked in a study diary.

### 2.4. Probiotic Composition

The multi-strain probiotic contained 6 × 10^9^ CFU of *Lactobacillus plantarum* Lp-115, 3 × 10^9^ CFU of *Lactobacillus bulgaricus* Lb-64, 18 × 10^9^ CFU of *Lactobacillus gasseri* Lg-36, 7.5 × 10^9^ CFU of *Bifidobacterium breve* Bb-03, 8 × 10^9^ CFU of *Bifidobacterium animalis sbsp. lactis* Bi-07, 7 × 10^9^ CFU of *Bifidobacterium bifidum* Bb-06, 450 × 10^6^ CFU of *Streptococcus thermophilus* St-21 and 45 × 10^6^ CFU of *Saccharomyces boulardii* DBVPG 6763, and excipients (40 mg microcrystalline cellulose, 5 mg silica, and 10 mg magnesium stearate) per capsule. The probiotic combination provided an assemblage of 5 × 10^10^ CFU/dose packaged in a capsule. The product was stored in the refrigerator at 4–6 °C at the clinical setting, and all the participants were instructed to store the product in the refrigerator at all times.

### 2.5. Outcome Measures

Primary outcome: fasting plasma glucose. Secondary outcomes: circulating HbA1c, triglycerides, free fatty acids, total cholesterol, HDL-c, LDL-c, hs-CRP, lipopolysaccharide, zonulin, SCFAs as well as insulin resistance, anthropometric measurements (body weight, body fat, and waist and hip circumferences), blood pressure, probiotic safety, gastrointestinal symptoms, and fecal metagenomic profiles.

### 2.6. Blood Sample Collection

Blood samples were collected after fasting overnight at baseline and at 12 weeks. The participants who did not have T2DM had a 75 g oral glucose tolerance test (OGTT) to assess their circulating glucose and insulin levels (at 0 min, 60 min and 120 min). Insulin resistance was calculated by the Insulin Sensitivity Index of Matsuda (ISI-M) [30] and the Homeostatic Model Assessment for Insulin Resistance (HOMA-IR) [31] using the glucose and insulin results from the OGTT and fasting test. Thus, the ISI-M data were missing for those previously diagnosed with T2DM.

The fasting plasma glucose, insulin, HbA1c, lipids, and hs-CRP were analysed at a medical laboratory (DHM Pathology). The plasma lipopolysaccharide (LPS) and zonulin were analysed as previously described [28].

### 2.7. SCFA Extraction and Analysis

SCFA extraction and gas chromatography-mass spectrometry (GC-MS) analysis of plasma were performed according to Skoglund et al. [32]. Acetic acid-d4 was used as an internal standard. The SCFAs were quantified using an Agilent 7890A gas chromatography system coupled with an Agilent 5975C inert XL EI/CI mass spectrometric detector (MSD, Agilent Technologies, Sydney, Australia). The identification of propionate, isobutyrate, butyrate, and isovalerate was based on the retention time of standard compounds and quantified using Agilent Mass Hunter Quantitative software. The quantification of each SCFA was based on the calibration curves obtained from increasing concentrations of standards diluted in ethyl acetate (500, 2000, and 4000 µM). All the calibration curves had an R^2^ ≥ 0.99.

### 2.8. Metagenomic Analysis of Fecal Microbiome

Stool samples were collected at baseline and at 12 weeks, as previously described [28]. Genomic DNA was isolated from 200 mg of stool sample using the QIAamp DNA stool kit (Qiagen), following the manufacturer’s protocol. Shotgun metagenomic libraries were generated using 1 ng of DNA and the Nextera XT protocol, according to the manufacturer’s instructions (Illumina). The libraries were pooled and 150 bp paired-end shotgun sequencing was performed using the Illumina HiSeqX platform. The metagenomic reads were quality filtered using SolexaQA++ with a Phred score > 20 [33]. After read trimming, the samples which did not meet the quality control criteria were discarded. In addition, only subjects who had samples from both baseline and week 12 were used for metagenomic analyses, resulting in 28 samples in the probiotic group (*n* = 14) and 42 samples in the placebo group (*n* = 21). Taxonomic information was assigned based on marker genes using MetaPhlAn2 [34]. Functional annotation was obtained using the HUMAnN2 pipeline with UniRef 50 [35]. In order to assess the microbiome beta-diversity, a principal coordinate analysis (PCoA) with bray-curtis distance was calculated using the vegan package (version 2.5-4) in R [36].

### 2.9. Probiotic Safety and Gastrointestinal Symptoms Assessment

The probiotic safety was determined by the incidence of adverse events over the study period of 12 weeks. The gastrointestinal symptoms were assessed via the Gastrointestinal Symptom Rating Scale (GSRS) [37].

### 2.10. Statistical Analysis

The calculation of the study sample size included a type I error of 0.05 and type II error of 0.20 (power = 80%). The number of participants needed to detect a mean difference in fasting plasma glucose of 2 mmol/L was 32. To reduce the bias of having two cohorts (participants with prediabetes and early T2DM) and no stratification at randomization, the sample was inflated to 30 per group [28]. An independent researcher prepared a simple randomization list, and the participants were then randomised to the probiotic or placebo groups using computer-generated random numbers (FileMaker Pro).

An intention-to-treat analysis was adopted, and the missing data were imputed with baseline values for a conservative estimate (i.e., no change). All the data were checked for normal distribution using the Shapiro–Wilk test. Descriptive statistics were presented as the mean ± standard deviation or median with interquartile range (IQR), as appropriate. The primary outcome (fasting plasma glucose) and some secondary outcomes (HbA1c and insulin resistance) were analysed using generalised linear models, and the following covariates were added to the model: sex, age, and baseline values. The remaining secondary outcomes were analysed using a one-way ANOVA or Kruskal Wallis test to compare the study groups at week 12. A repeated measures ANOVA using the Bonferroni correction or Wilcoxon matched-pairs signed rank test was used to analyse the differences between the baseline and endpoint values. A planned subgroup analysis was performed in participants taking metformin within the probiotic and placebo groups. Statistical analyses were performed using SPSS Statistics version 22 (IBM, Australia).

For the intestinal microbiome analysis, statistical tests were performed in R [38]. In order to compare the effect of treatment between two groups on taxonomic and functional abundances, the Wilcoxon matched-pairs signed rank test was used. A Spearman correlation analysis using clinical data and species abundances at week 12 was also performed.

## 3. Results

### 3.1. Study Participants

**Figure 1:**
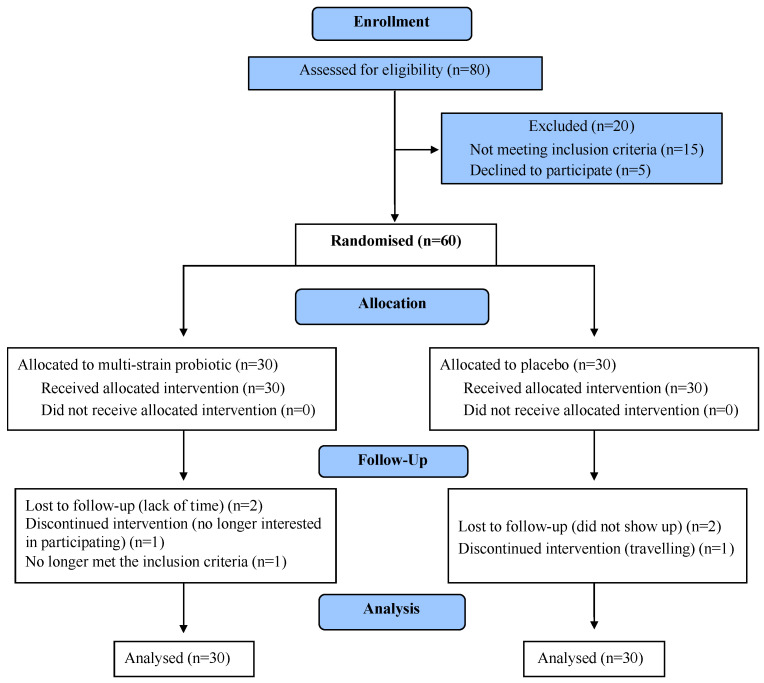
CONSORT flowchart of participants’ progress through the study.

The participant recruitment was from August 2015 to July 2016. The participants’ progression through the trial is presented in Figure 1 (Consolidated Standards of Reporting Trials, CONSORT diagram [39]). Overall, 88% of the participants completed the study. The baseline characteristics of those who completed the study and those lost to follow-up were similar. The baseline characteristics of the participants included in the analysis are shown in Table 1.

Twenty-eight participants treated with metformin participated in the study. The duration on this medication varied from ≤10 days to ≥10 years before starting the study. Among the six participants who started taking metformin in the previous 10 days, three were in the probiotic group. The daily dosage of metformin used varied from 500 to 3000 mg. In the probiotic group, the participants were taking 850 (*n* = 1), 1000 (*n* = 9), 2000 (*n* = 3), and 3000 mg (*n* = 1) of metformin per day. In the placebo group, the participants were taking 500 (*n* = 4), 1000 (*n* = 8), and 2000 mg (*n* = 2) of metformin per day.

### 3.2. Probiotic Detection and Quality Control

The target probiotic species composition was validated by shotgun metagenomic sequencing. Two probiotic capsules were analysed. The relative abundance for each of the species in the capsules were calculated using the MetaPhlAn2 pipeline [34]. The detected relative abundance of each species in both capsules was close to the designed targets in the laboratory (supplementary Figure S1A). After the 12-week intervention, the probiotic species *Bifidobacterium animalis*, *Bifidobacterium bifidum*, and *Bifidobacterium breve* were detected in 0.01% ± 0.02%, 0.22% ± 0.62%, and 0.16% ± 0.32%, respectively, of fecal samples from participants treated with the multi-strain probiotic (supplementary Figure S1B)

### 3.3. The Effect of the Multi-Strain Probiotic in Metabolic and Inflammatory Markers

There was no difference in the fasting plasma glucose (regression coefficient: 0.04; 95% CI: −0.41, 0.48; *p* = 0.48), HbA1c (regression coefficient: 0.04; 95% CI: −0.21, 0.29; *p* = 0.73), HOMA-IR (regression coefficient: 0.72; 95% CI: −0.35, 1.79; *p* = 0.18), or ISI-M (regression coefficient: 1.47; 95% CI: −4.43, 7.37; *p* = 0.62) and no differences in the anthropometric measurements, lipids, blood pressure, or inflammatory markers between the probiotic and placebo groups. A sub-group analysis in participants taking metformin found a decrease in the fasting plasma glucose (F [1, 10] = 5.07, *p* = 0.048, ηp2 = 0.34), HbA1c (F [1, 10] = 5.90, *p* = 0.036, ηp2 = 0.37), HOMA-IR (F [1, 10] = 6.11, *p* = 0.033, ηp2 = 0.38), fasting plasma insulin (F [1, 10] = 5.46, *p* = 0.044, ηp2 = 0.38), and zonulin (F [1, 10] = 5.10, *p* = 0.048, ηp2 = 0.34) between the baseline and week-12 values in those in the probiotic group but not the placebo group. An increase in insulin sensitivity (measured by ISI-M) was found in participants taking metformin in the probiotic group (*p* = 0.046) but not in the placebo group. The baseline and after-treatment glycaemic parameters and insulin sensitivity indices are reported in Table 2. The remaining outcome measures are reported in supplementary Table S1.

### 3.4. Probiotic Safety

A total of four adverse events and no serious adverse events were reported during the study. Three participants with T2DM and one with prediabetes, all in the probiotic group, reported mild lower-limb infection (fungal and cellulitis), and one participant in the placebo group reported a urinary tract infection. The plasma hs-CRP levels in these participants were above the normal range at baseline and remained high during and after the intervention.

### 3.5. Gastrointestinal Symptoms

No significant difference in the overall GSRS score and in the severity of diarrhoea, nausea, flatulence, and abdominal pain was found between the probiotic and placebo groups after intervention (22.1 ± 8.1 vs. 22.7 ± 7.7, *p* = 0.68; 1.3 ± 0.8 vs. 1.4 ± 1.3, *p* = 0.73; 1.3 ± 0.9 vs. 1.2 ± 0.5, *p* = 0.89; 1.4 ± 0.8 vs. 1.9 ± 1.4, *p* = 0.13; 1.3 ± 0.7 vs. 1.6 ± 1.4, *p* = 0.44, respectively).

### 3.6. Characterization of the Intestinal Microbiota

**Figure 2:**
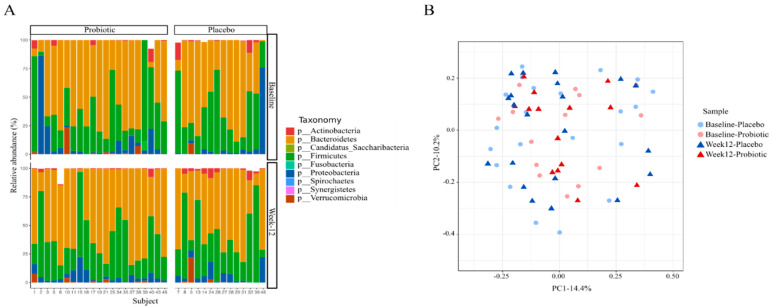
Shift in intestinal microbial profile after the 12-week intervention. (**A**) Microbial profile at the phylum level in each groups and timeline. (**B**) Principal coordinate analysis (PCoA) of Bray–Curtis distances at the species level between the intestinal microbial communities of subjects in each group.

The baseline intestinal microbial profile showed that 96.5% of the sequences were distributed among three bacterial phyla—namely, *Bacteroidetes*, *Firmicutes*, and *Proteobacteria* (31.1%, 56.8%, and 8.5%, respectively; Figure 2a). The remaining 3.5% were occupied mainly by *Actinobacteria* and *Verrucomicrobia* (1.8% and 1.3%, respectively). In the whole cohort, the *Firmicutes* to *Bacteroidetes* ratio was 2.60. Moreover, the participants shared 14% of all the detected families and genera and 3% of all the detected species. This shows the variability of the intestinal microbiota among participants. No significant differences in the bacterial beta diversity at the species level were found between the probiotic and placebo groups at baseline and week 12 (ANOSIM test: *p* = 0.507 and 0.485, respectively; Figure 2b). Also, a subgroup analysis in participants on metformin showed the probiotic did not have a significant effect on the bacterial beta diversity at the species level (ANOSIM test: *p* = 0.97) (supplementary Figure S2). An analysis of the relative abundance at the species level revealed shifts in some microbial members in the probiotic and placebo groups after the 12-week intervention (Table 3).

### 3.7. Intestinal Microbial Species Correlated with Clinical Outcomes

**Figure 3:**
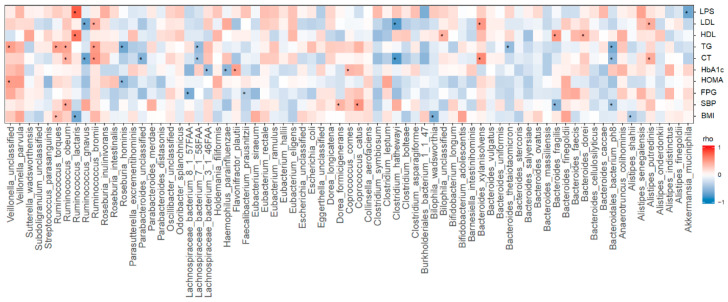
Spearman correlation analysis between species abundance and clinical outcomes (* *p* < 0.05). LPS: lipopolysaccharide; LDL: low-density lipoprotein; HDL: High-density lipoprotein; TG: triglycerides; CT: Total Cholesterol; HbA1c: Hemoglobin A1c; HOMA: Homeostatic Model Assessment; FPG: Fasting Plasma Glucose; SBP: Systolic Blood Pressure; BMI: body mass index.

A Spearman correlation analysis using clinical data and species abundances at week 12 was performed (*n* = 35). In total, 26 microbial species with relative abundances >0.01% were significantly correlated with the BMI, fasting plasma glucose, HbA1c, HOMA, HDL, LDL, systemic blood pressure (Figure 3).

### 3.8. The Impact of the Multi-Strain Probiotic on SCFA Profiles and Metabolic Pathways

**Figure 4:**
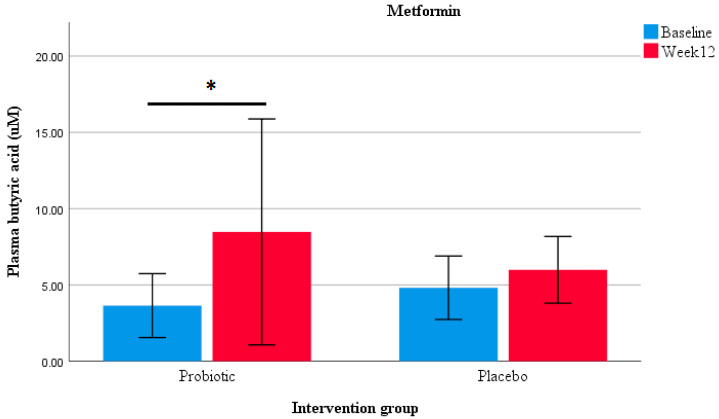
Plasma butyrate concentration. Box plots representing the effect on butyrate levels in participants taking metformin in the probiotic (*n* = 14) and placebo (*n* = 14) groups. * Wilcoxon matched-pairs signed rank test showing significant differences within the probiotic group (*p* < 0.05).

An analysis between the baseline and week 12 values determined an increase in plasma butyrate concentrations in participants in the probiotic group (*p* = 0.039) and in those taking metformin within the probiotic group after the 12-week intervention (*p* = 0.038; Figure 4). No differences in SCFA concentrations were found between the probiotic and placebo groups (supplementary Table S2). Moreover, a correlation analysis showed that plasma butyrate and propionate were negatively associated with the HOMA-IR index, LDL-cholesterol, and LPS levels (butyrate: r = −0.56, *p* < 0.05; r = −0.57, *p* = 0.01; r = −0.46, *p* < 0.05 and propionate: r = −0.54, *p* < 0.05; r = −0.66, *p* < 0.01; r = −0.58, *p* = 0.01, respectively).

The functional annotation of metagenomic reads from all the samples indicated an enrichment of the pyruvate fermentation to butanoate, and Bifidobacterium shunt pathways were found in participants taking metformin in the probiotic group in comparison to those in the placebo group (Wilcoxon, *p* = 0.03 vs. *p* = 0.81 and *p* = 0.03 vs. *p* = 0.27, respectively, supplementary Figure S3).

## 4. Discussion

The study showed that the administration of a multi-strain probiotic for 12 weeks had no overall effect on metabolic and systemic inflammatory markers in subjects with prediabetes and recently diagnosed with T2DM. However, within the probiotic and metformin subgroup a decrease in fasting plasma glucose, insulin resistance, and zonulin protein was observed, but not within the placebo group. The probiotic alone and in combination with metformin increased the plasma butyrate concentrations and SCFA-producing bacteria after the 12-week intervention.

Different questions arise from the investigations assessing the efficacy of probiotics. These include the impact of different formulations, dosages, and length of supplementation on the host’s response to the treatment. Therefore, these aspects were considered during the design of this study. The selection of the eight probiotic strains/species was based on clinical studies showing these strains/species affected on one or multiple health outcomes in participants with obesity, insulin resistance, and T2DM (i.e., body weight, glycaemia, lipid profile, and inflammatory markers) [40,41]. The total dosage administered was 2 × 10^11^ CFU/day (based on evidence suggesting that doses ≥ 1 × 10^11^ CFU/day of probiotics had a greater effect on blood glucose than lower doses in participants with T2DM [40]). The probiotic was administered for 12 weeks, as previous reports showed that probiotic supplementation for ≥8 weeks improved metabolic and inflammatory markers in obesity and T2DM [40,41]. However, in the current study the multi-strain probiotic did not improve the clinical outcome measures.

Research evidence from human intestinal microbial profiles demonstrates that each individual has a unique intestinal bacterial composition (in diversity and abundance). Nevertheless, it has been suggested that healthy subjects share similarities in the composition and functional gene profiles (core microbiome), which may help maintain healthy gut function [42]. It is also known that deviations from a core microbiome may be associated with different physiological or pathological states, such as obesity and T2DM [43,44]. A few studies have reported the relationship between the gut microbiota composition and T2DM. Larsen et al. [14] showed that the ratio of *Bacteroidetes* to *Firmicutes* was correlated with elevated plasma glucose concentrations but not with body mass index in men with T2DM compared to non-diabetic controls. In this study, the *Firmicutes* to *Bacteroidetes* ratio was 2.60, a result that was similar to that reported by Larsen et al., in which a ratio lower than 3.3 (reported in lean controls) was found in subjects with T2DM (0.47). Moreover, in a metagenome-wide association study and 16S rRNA gene-based investigations of the fecal microbiota of participants with T2DM, the intestinal microbiome was characterised by a moderate degree of dysbiosis; a decrease in the abundance of SCFA-producing bacteria, such as those of the *Faecalibacterium*, *Bifidobacterium*, and *Akkermansia* genera; and an increase in pro-inflammatory bacteria [45,46]. In general, the *Bifidobacterium* genus contributes to gut homeostasis and host health by producing acetates and lactates during carbohydrate fermentation, which can be converted into butyrate by other intestinal bacteria through cross-feeding interactions [47]. The current study showed an increase in the relative abundance of *Bifidobacterium breve* and other acetate-producing bacteria, including *Akkermansia muciniphila* and *Clostridium hathewayi* (cluster XIVa) in the probiotic group. The probiotic also tended to decrease the pro-inflammatory bacteria *Prevotella copri*. However, no significant differences in bacterial beta diversity at the species level were found between the probiotic and placebo groups at 12 weeks.

The administration of *A. muciniphila* has been shown to restore intestinal mucus-layer thickness, maintain bacterial homeostasis, and improve high-fat diet-induced metabolic disorders in obese mice by decreasing weight gain, insulin resistance, serum triglycerides, and hepatic inflammation [12,48,49]. These anti-obesity effects are potentially due to an increase in energy expenditure as well as fecal energy excretion, which has been associated with a higher intestinal epithelial cell turnover and a reduction in carbohydrate absorption [50]. In addition, *A. muciniphila* supplementation has shown to decrease metabolic endotoxaemia, which is characterised by high circulating LPS levels and linked with the development of T2DM [51,52]. The current study showed that *A. muciniphila* was negatively correlated with plasma LPS levels (*p* < 0.05) but not with other metabolic markers. Additionally, even though an increase in the relative abundance of *A. muciniphila* was found in the probiotic group, there were no differences in the plasma LPS levels between the probiotic and placebo groups. As a probiotic, the anti-obesity and anti-diabetic effects of *A. muciniphila* reported in animal studies need to be confirmed in participants with metabolic disorders.

SCFA production and/or regulation has been proposed as one of the mechanisms by which probiotics promote health outcomes [53]. An analysis between the baseline and week-12 values determined an increase in plasma butyrate concentrations in participants in the probiotic group (*p* = 0.039) and in those taking metformin in the probiotic group after the 12-week intervention (*p* = 0.038). Furthermore, an increase in the abundance of metabolic pathways associated with the production of butyrate was found in participants taking metformin combined with the probiotic, but not in those taking probiotic or metformin alone (*p* < 0.05). Along with these findings, the abundance of the acetate and butyrate producer *Anaerotruncus colihominis* (Cluster IV) was increased only in participants taking both metformin and the probiotic. In participants taking either probiotic or metformin alone, other acetate-producing bacteria showed an increase in abundance, including *Bifidobacterium breve* and *Dorea logicatena* (Cluster XIVa). In terms of clinical outcomes, the restoration of the diversity and abundance of SCFA-producing bacteria has been associated with improved HbA1c levels in participants with T2DM [19]. The current study showed a decrease in glycaemia and insulin resistance in participants taking metformin and the probiotic, but not in those taking metformin and the placebo. To the best of our knowledge, this is the first study to suggest a possible beneficial interaction between a multi-strain probiotic and metformin in patients with prediabetes or T2DM as a combined therapy.

Positive correlations between zonulin, a marker and modulator of intestinal permeability; insulin resistance; and increased inflammatory and glycaemic markers have been reported in subjects with obesity [54] and T2DM [55]. In the current study, zonulin levels in plasma that were above average range at baseline (>200 mg/dL) decreased significantly to normal levels at week 12 in participants taking metformin and the probiotic. The effect of metformin in combination with probiotics on this permeability marker has not been previously assessed—however, probiotics’ protective effect on the intestinal barrier has been reported in previous studies [56,57]. Since butyrate has been shown to maintain gut barrier integrity [58], it is possible that the zonulin levels were altered by the probiotics and metformin, encouraging the intestinal microbiome to increase the production of luminal butyrate.

The study’s limitations include the low number of visits during the study—a potential risk factor for non-compliance. Even though more than 70% of the participants were considered compliant (taking ≥80% of the study products) and no one took less than 50% of the recommended dosage, further long-term studies with additional follow-up visits to increase adherence to the treatment regimens (i.e., probiotic and lifestyle interventions) are required to confirm the outcomes of this study. In addition, more male participants were enrolled in the probiotic group than the placebo group, which might limit the interpretation of the results. To reduce the between-group bias, the fasting plasma glucose and HbA1c and insulin resistance were analysed using generalised linear models, with gender, age, metformin, and baseline values as covariants. No male preponderance was found in the subgroup analysis performed in those taking metformin. An additional aspect to consider was that only 47% and 70% of the fecal samples in the probiotic and placebo groups, respectively, were used for metagenomic analysis, and this difference may have limited the analyses and outcomes. The remaining samples were either not provided or discarded due to quality-related procedures. Finally, it is known that probiotics do not recolonise the intestinal tract. Rather, they are transient colonisers, and the wash-out of administered doses can take approximately 4–6 weeks, thereby leading to a therapy that may not be permanent. This implies that for lasting or perhaps significant effects, probiotics need to be taken for a longer term.

## 5. Conclusions

The administration of the multi-strain probiotic with or without metformin was shown to be safe and well tolerated in this pilot study. Although no significant changes in metabolic, inflammatory and permeability markers between the probiotic and placebo groups were observed, significant improvements in the fasting plasma glucose, insulin resistance, and permeability marker zonulin were found in the participants taking metformin and the probiotic together, with beneficial shifts in SCFA-producing bacteria. This study provided suggestive pilot data that probiotics may enhance the efficacy of metformin and regulate butyrate production in those with prediabetes and recently diagnosed T2DM. The findings reported partly clarify the possible mechanisms by which probiotics may assist as an adjunct supplement to metformin in the management of individuals with high blood glucose levels.

## 6. Patents

Patent application numbers held by Medlab Clinical include: 2015258769; 2-17101478; PCT/AU2018/051183; PCT/AU2018/051182.

## Figures and Tables

**Table 1:** Baseline characteristics of participants by treatment group.

	*Probiotic*	*Placebo*
	(*n* = 30)	(*n* = 30)
Female	13 (43%)	19 (63%)
Male	17 (57%)	11 (37%)
Age (years)	61.4 ± 8.9	56.1 ± 12.3
Prediabetes	17	19
T2DM	13	11
Metformin	14 (47%)	14 (47%)
Lipid-lowering medication ^a^	15 (50%)	8 (27%)
BP-lowering medication ^b^	13 (43%)	14 (47%)
Height (m)	1.7 ± 0.1	1.7 ± 0.1
Body weight (kg)	100.1 ± 20.4	101.7 ± 21.9
BMI (kg/m^2^)	35.5 ± 6.2	36.3 ± 7.5

Results are mean ± SD or number and percentage where appropriate. BP: blood pressure. ^a^ Statins and ezetimibe. ^b^ ACE inhibitors, angiotensin II receptor blockers, beta-blockers, and calcium channel blockers.

**Table 2:** Glycaemic parameters and insulin sensitivity indices by intervention group and within participants taking metformin.

		All Participants		Participants on Metformin	
Parameter	Time-Point	Probiotic (*n* = 30)	Placebo (*n* = 30)	Probiotic (*n* = 14)	Placebo (*n* = 14)
FPG (mmol/L)	*Baseline*	5.9 ± 0.8	5.7 ± 0.6	8.6 ± 4.5	6.9 ± 2.3
	*Week 12*	5.7 ± 0.6	5.8 ± 0.7	7.8 ± 4.3 ^a,^*	6.7 ± 1.8
HbA1c (%)	*Baseline*	6.1 ± 0.6	5.9 ± 0.4	7.3 ± 1.7	6.6 ± 1.4
	*Week 12*	5.9 ± 0.5	6.0 ± 0.3	6.8 ± 1.7 ^a,^*	6.5 ± 1.1
HOMA-IR	*Baseline*	3.4 ± 1.9	3.3 ± 1.8	5.0 ± 4.7	3.5 ± 2.2
	*Week 12*	2.7 ± 1.5	3.3 ± 3.3	3.5 ± 3.5 ^a,^*	3.2 ± 2.5
FPI (mU/L)	*Baseline*	12.8 ± 6.5	13.4 ± 6.9	12.1 ± 5.8	11.9 ± 5.8
	*Week 12*	10.4 ± 5.4	12.4 ± 11.2	8.6 ± 4.1 ^a,^*	11.0 ± 6.9
ISI-M	*Baseline*	3.9(2.6)	2.5(1.9)	3.2(4.8)	3.9(1.5)
	*Week 12*	3.6(6.0)	3.8(3.0)	6.1(8.0) ^b,^*	4.3(6.0)

Data are means ± SD or median (IQR). *p* values are obtained from a repeated measures ANOVA ^a^ or a Wilcoxon matched-pairs signed rank test ^b^ for within-group comparisons. * *p* < 0.05. FPG: Fasting Plasma Glucose; FPI: Fasting Plasma Insulin; ISI-M: Insulin sensitivity index-Matsuda; HOMA-IR: Homeostatic Model Assessment-Insulin Resistance

**Table 3:** Changes in relative abundance at the species level by intervention group and within participants taking metformin.

	All Participants		Participants on Metformin	
Species	Probiotic	Placebo	Probiotic	Placebo
*Bifidobacterium breve*	↑ *		↑ ^a^	
*Bacteroides caccae*	↑ *		↑ ^a^	↑ *
*Bacteroidales bacterium* ph8	↑ *			
*Akkermansia muciniphila*	↑ ^a^			
*Clostridium hathewayi*	↑ ^a^			
*Prevotella copri* ^a^	↓ ^a^			
*Flavonifractor plautii*	↓ ^a^			
*Bacteroides faecis*		↑ *		
*Bacteroides finegoldii*		↑ *		
*Bacteroides salyersiae*		↑ *		
*Bacteroides thetaiotaomicron*		↑ *		
*Parabacteroides merdeae*		↑ *		
*Bilophila wadsworthia*		↑ **		
*Desulfovibrio desulfuricans*		↓ *		
*Bacteroides uniformis*			↓ *	
*Anaerotruncus colihominis*			↑ ^a^	
*Dorea formicigenerans*				↑ *
*Dorea longicatena*				↑ *
*Lachnospiraceae bacterium*				↑ *

*p* values were obtained from Wilcoxon matched-pairs signed rank test (^a^ *p* = 0.05; * *p* < 0.05; ** *p* < 0.005).

## Supplementary Materials

The following are available online at https://www.mdpi.com/2072-6643/12/7/2041/s1: Table S1: Outcome measures by treatment group and within participants taking metformin. Table S2: Plasma SCFA concentrations by treatment group within the participants on metformin. Figure S1: Probiotic detection and quality control. Figure S2: PCoA of Bray-Curtis distances between intestinal microbial communities of probiotics and placebo group in Metformin treated group. Figure S3: SCFA-producing pathways abundance by intervention group and within participants on metformin.
