# Supplementary material for: Targeting the Intestinal Microbiota to Prevent Type 2 Diabetes and Enhance the Effect of Metformin on Glycaemia: A Randomised Controlled Pilot Study
Source: Nutrients. 2020 Jul 9;12(7):2041. doi: 10.3390/nu12072041 (PMC7400852; doi:10.3390/nu12072041)
Supplement: Supplementary file 1 [file nutrients-12-02041-s001.pdf]

Supplementary information

Table S1. Outcome measures by treatment group and within participants taking metformin.

		All Participants		Participants on metformin	
**Parameter**	**Time-point**	**Probiotic (N=30)**	**Placebo (N=30)**	**Probiotic (N=14)**	**Placebo (N=14)**
Weight (kg)	*Baseline*	100.1±20.4	101.7±21.9	97.6±21.9	101.0±20.6
	Week 12	97.5±21.2	100.8±22.8	91.7±23.9	99.5±22.2
BMI (kg/m^2)	*Baseline*	35.5±6.20	36.3±7.5	35.0±6.1	35.6±8.2
	Week 12	34.5±6.3	36.0±7.8	32.3±6.5	35.1±8.8
Waist (cm)	*Baseline*	114.7±12.9	114.3±14.7	113.7±13.6	113.4±12.8
	Week 12	114.3±16.0	116.0±17.1	109.4±18.0	116.1±16.2
Waist:Hip	*Baseline*	0.97±0.06	0.95±0.07	1.0±0.1	1.0±0.1
	Week 12	0.97±0.06	0.98±0.72	0.9±0.1	1.0±0.1
Body fat (%)	*Baseline*	37.7±8.2	41.8±7.9	39.5±9.1	40.3±7.8
	Week 12	38.1±7.8	40.9±8.4	37.3±8.5	38.9±8.7
Systolic BP (mmHg)	*Baseline*	133.0±10.8	127.8±12.5	134.5±14.4	130.4±12.3
	Week 12	128.5±12.5	127.0±13.1	129.0±17.9	127.5±11.3
Diastolic BP (mmHg)	*Baseline*	79.8±7.3	81.6±6.3	79.4±6.5	83.7±5.8
	Week 12	78.5±5.8	81.6±7.3	78.0±6.6	82.6±6.1
TC (mmol/L)	*Baseline*	4.9±1.1	5.4±1.3	4.7±1.0	5.1±1.1
	Week 12	4.9±1.1	5.3±1.4	4.4±0.9	4.7±0.8
HDL-c (mmol/L)	*Baseline*	1.2±0.3	1.3±0.2	1.3±0.3	1.3±0.2
	Week 12	1.2±0.3	1.2±0.2	1.2±0.3	1.2±0.2
LDL-c (mmol/L)	*Baseline*	2.8±1.1	3.2±1.0	2.7±0.9	3.0±1.1
	Week 12	2.9±1.1	3.2±1.1	2.4±1.0	2.7±0.8
Triglycerides (mmol/L)	*Baseline*	1.8±0.9	1.9±1.2	1.7±0.8	1.6±0.8
	Week 12	1.7±0.7	2.1±1.3	1.6±0.9	1.6±0.6
FFA (μmol/L)	*Baseline*	608.5±296.4	647.8±234.9	687.1±345.8	651.1±248.4
	Week 12	618.0±219.1	642.0±183.2	714.6±195.2	621.6±229.3
hs-CRP (mg/L)	*Baseline*	3.9±4.1	3.0±2.4	4.7±4.1	5.5±9.1
	Week 12	4.1±4.7	4.3±6.2	5.4±5.8	5.5±10.3
LPS (EU/mL)	*Baseline*	0.5±0.2	0.6±0.3	0.5±0.3	0.5±0.3
	Week 12	0.5 + 0.2	0.5±0.2	0.5±0.2	0.5±0.2
Zonulin (mg/dL)	*Baseline*	203.6±171.9	207.8±172.9	291.0±211.3	233.9±239.0
	Week 12	166.7 ± 112.6	136.3±79.9	197.9±119.3[a]*	151.6±71.8

Data are means ± SD or median (IQR). Difference between baseline and endpoint. P value obtained from repeated measure ANOVA[a] or Wilcoxon matched-pairs signed rank test[b] for the within-group comparisons. *$p < 0.05$. BMI: body mass index; BP: blood pressure; TC: total cholesterol; HDL-c: high-density lipoprotein cholesterol; LDL: low-density lipoprotein cholesterol; FFA: free fatty acids; hs-CRP: high-sensitive C-reactive protein; LPS: lipopolysaccharide.

Table S2. Plasma SCFA concentrations by treatment group within the participants on metformin

		All participants		Participants on metformin	
**SCFA (μM)**	**Time-point**	**Probiotic (N=30)**	**Placebo (N=30)**	**Probiotic (N=13)**	**Placebo (N=14)**
Propionate	Baseline	60.7 (70.0)	56.6 (85.2)	50.2 (57.4)	56.6 (83.8)
	Week 12	89.2 (79.8)	99.6 (80.1)	85.3 (80.3)	99.6 (81.6)
Isobutyrate	Baseline	6.1 (8.4)	6.0 (11.2)	5.4 (6.9)	6.0 (6.0)
	Week 12	6.9 (17.0)	7.0 (11.7)	6.6 (9.8)	7.0 (7.1)
Butyrate	Baseline	4.3 (4.9)	3.5 (7.3)	3.2 (4.3)	3.5 (5.3)
	Week 12	5.8 (8.3) [a*]	6.5 (7.8)	6.3 (9.1)[a*]	6.9 (4.6)
Isovalerate	Baseline	0.4 (2.2)	0.1 (2.1)	0.0 (1.1)	0.1 (1.6)
	Week 12	0.9 (4.7)	1.2 (3.6)	1.4 (3.0)	1.1 (5.8)
Total	Baseline	72.3 (86.6)	62.9 (111.1)	60.2 (67.3)	62.9 (95.6)
	Week 12	103.3 (113.3)	120.4 (99.3)	100.0 (102.4)	114.5 (98.2)

Data are median (IQR). [a]Difference between baseline and endpoint. P value obtained from Wilcoxon matched-pairs signed rank test for the within-group comparisons. *$p < 0.05$

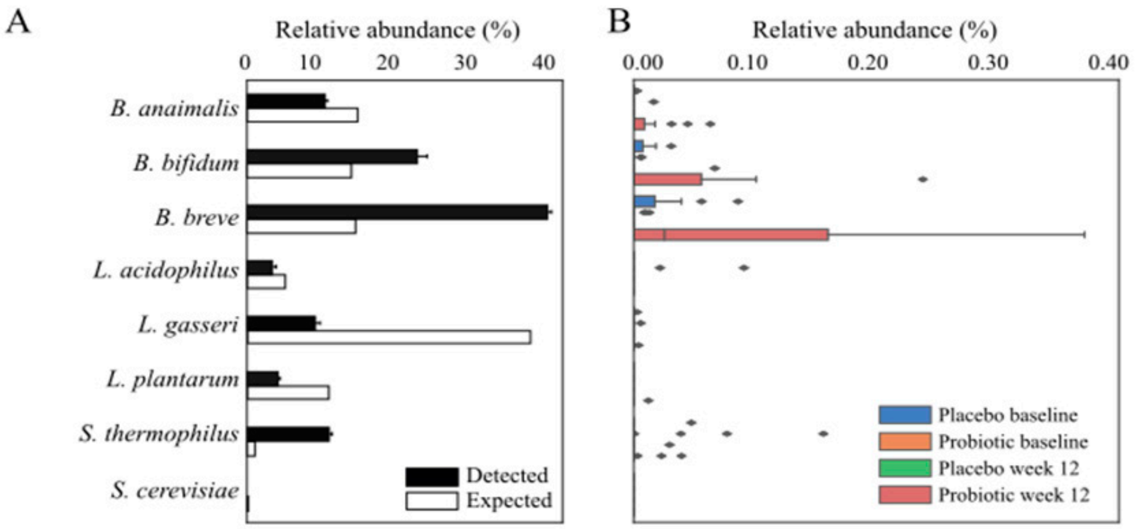

Figure S1. Probiotic detection and quality control. **A.** Comparison of the relative abundance of species in the capsule between the target formulation (Expected) and shotgun metagenomic sequencing (Detected, n=2). **B.** Relative abundance of probiotic species found in the study groups at baseline and after the 12-week intervention.

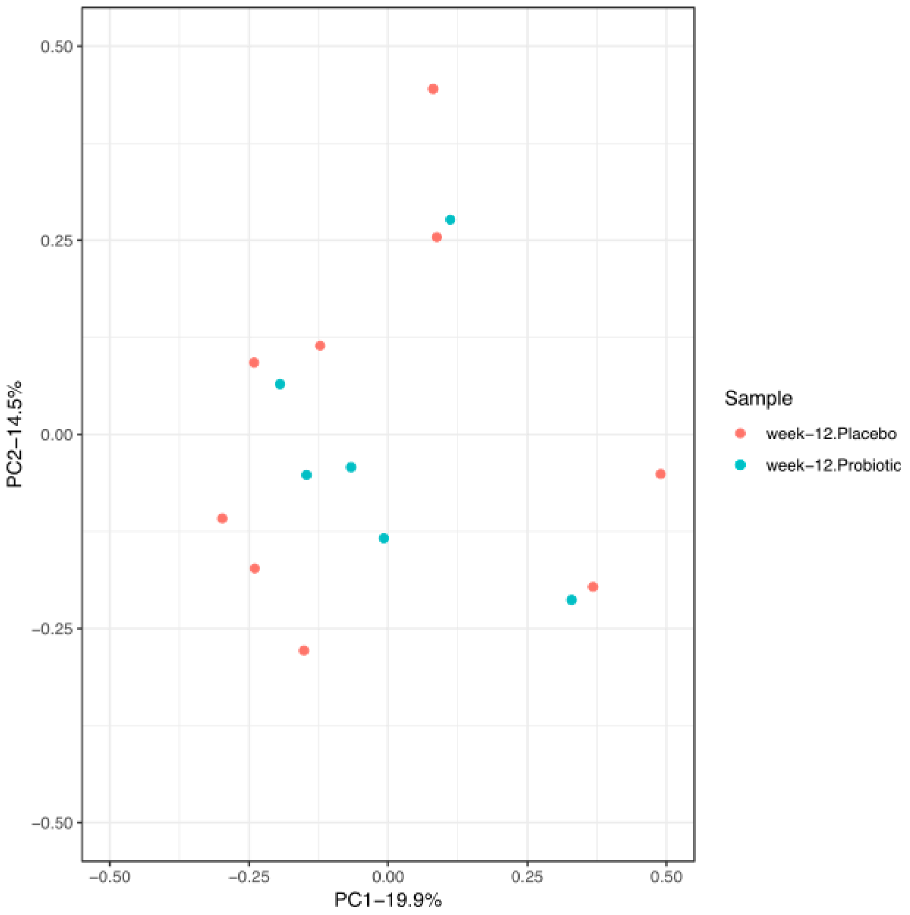

Figure S2. PCoA of Bray-Curtis distances between intestinal microbial communities of probiotics and placebo groups in Metformin treated group after 12 weeks of intervention.

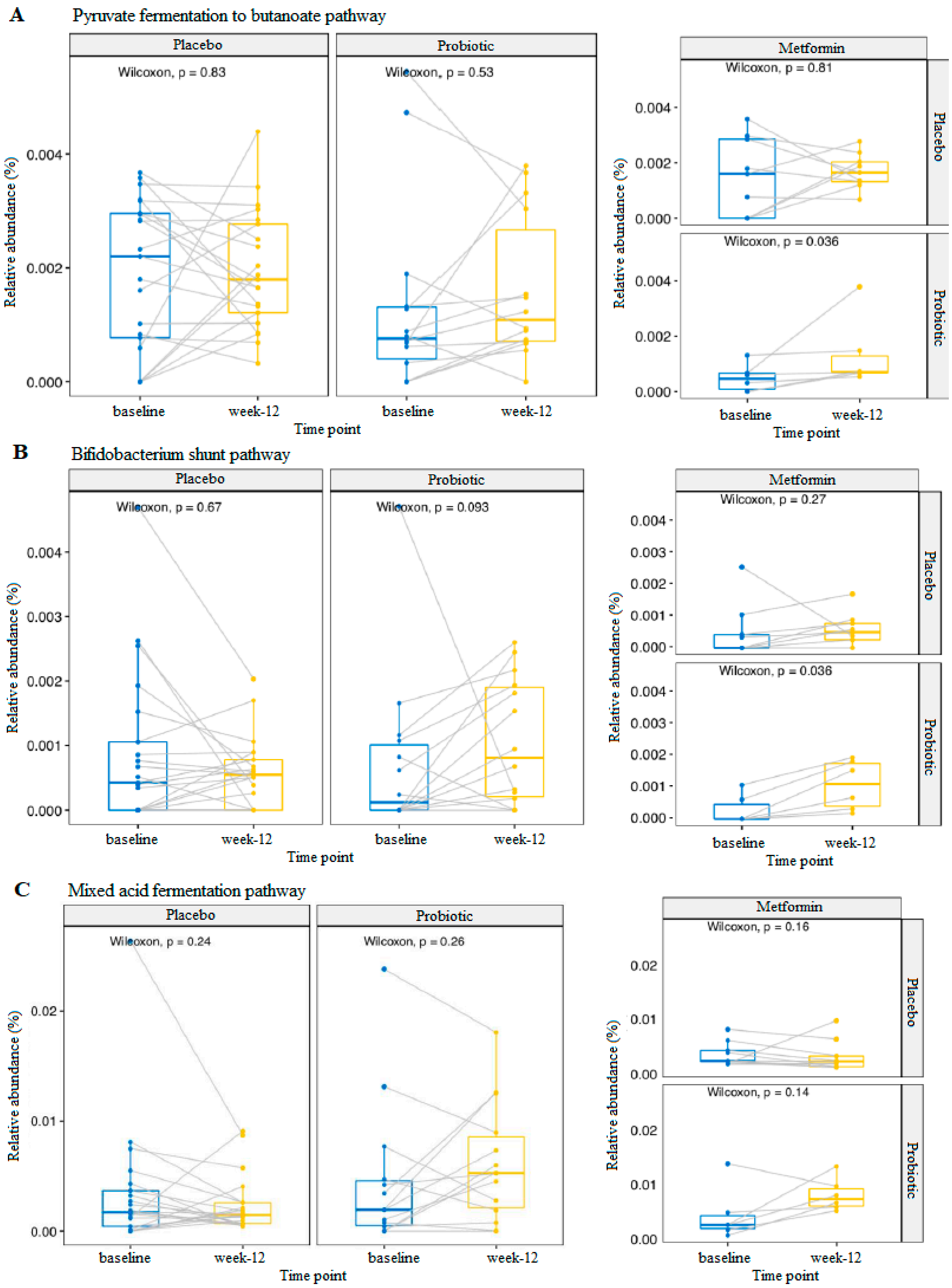

Figure S3. Relative abundance of SCFA-producing pathways in the faecal microbiota of participants in the intervention and metformin groups.
